# Decentralized Investigation of Bacterial Outbreaks Based on Hashed cgMLST

**DOI:** 10.3389/fmicb.2021.649517

**Published:** 2021-05-28

**Authors:** Carlus Deneke, Laura Uelze, Holger Brendebach, Simon H. Tausch, Burkhard Malorny

**Affiliations:** Department Biological Safety, German Federal Institute for Risk Assessment, Berlin, Germany

**Keywords:** cgMLST, WGS typing, molecular surveillance, comparative microbial genomics, *Salmonella*

## Abstract

Whole-genome sequencing (WGS)-based outbreak investigation has proven to be a valuable method for the surveillance of bacterial pathogens. Its utility has been successfully demonstrated using both gene-by-gene (cgMLST or wgMLST) and single-nucleotide polymorphism (SNP)-based approaches. Among the obstacles of implementing a WGS-based routine surveillance is the need for an exchange of large volumes of sequencing data, as well as a widespread reluctance to share sequence and metadata in public repositories, together with a lacking standardization of suitable bioinformatic tools and workflows. To address these issues, we present *chewieSnake*, an intuitive and simple-to-use cgMLST workflow. *ChewieSnake* builds on the allele calling software chewBBACA and extends it by the concept of allele hashing. The resulting hashed allele profiles can be readily compared between laboratories without the need of a central allele nomenclature. The workflow fully automates the computation of the allele distance matrix, cluster membership, and phylogeny and summarizes all important findings in an interactive HTML report. Furthermore, *chewieSnake* can join allele profiles generated at different laboratories and identify shared clusters, including a stable and intercommunicable cluster nomenclature, thus facilitating a joint outbreak investigation. We demonstrate the feasibility of the proposed approach with a thorough method comparison using publically available sequencing data for *Salmonella enterica*. However, *chewieSnake* is readily applicable to all bacterial taxa, provided that a suitable cgMLST scheme is available. The workflow is freely available as an open-source tool and can be easily installed via conda or docker.

## Introduction

Whole-genome sequencing (WGS)-based typing approaches allow the highly discriminatory comparison of the similarity of bacterial genomes. The results are used for the investigation of disease outbreaks, source attribution, contamination control, and surveillance of bacterial pathogens (Franz et al., 2016; Ronholm et al., 2016; Jagadeesan et al., 2019). A number of sequence-based typing approaches exist, each with their own advantages and drawbacks (Uelze et al., 2020b). Generally, the analysis of single-nucleotide polymorphisms (SNPs) is considered the method with the highest resolution. In particular, the National Center for Biotechnology Information (NCBI) pathogen detection pipeline is an SNP-based molecular typing system for global microbial surveillance based on publically available sequencing data^1^. Another high-resolution typing approach is the core genome/whole-genome multilocus sequence typing (cg/wgMLST), which was derived from the concept of classic multilocus sequence typing (MLST), with the distinction that the initial seven-gene multi-locus scheme was expanded to hundreds or thousands of gene loci (Maiden et al., 2013). CgMLST is a gene-by-gene approach, which functions by aligning complete or draft genome assemblies to a scheme consisting of a set of loci and a collection of associated numbered allele sequences. One of the advantages of cgMLST is that no outbreak specific reference is required, and therefore, it is a suitable and unbiased method to identify possible clusters from samples from an entire species. During the allele calling step, each locus is searched in the assembly, and if it matches an existing allele sequence, the number of that allele is assigned. In the case that an allele sequence is not yet contained in the scheme, a new allele number is created, and the allele sequence is added for future inquiries. From the set of allele numbers for each locus, a so-called allele profile is derived. The similarity between two or several genomes is estimated by comparing their respective allele profiles and calculating the total number of different alleles. Allele differences are first determined pairwise, before a distance matrix is derived by cross-comparison for all samples. Finally, a phylogenetic tree can be computed from the distance matrix through various clustering techniques such as neighbor-joining, minimum-spanning trees, or hierarchical clustering. Large minimum-spanning trees can be visualized, e.g., with grapeTree^2^ (Zhou et al., 2018) or PHLYOViZ^3^ (Francisco et al., 2012).

Core genome multilocus sequence typing clustering results are dependent on the choice of cgMLST scheme, with the number and type of loci being of great importance. The loci for each scheme should be chosen carefully to account for the unique genetic background of different species. Generally, the chosen loci should be part of the core genome (hence the name) of a taxonomic group and as such be present in the majority of all isolates of this group. A number of cgMLST schemes for major species are curated by various (at times commercial) organizations and research groups, such as Enterobase^4^, Institut Pasteur^5^, Ridom SeqSphere+^6^, or chewBACCA^7^, and can often be obtained freely from their websites.

One major disadvantage of a classic numbered cgMLST allele sequence scheme is that results from different laboratories, even when using the same scheme, are not directly comparable. This is caused by the fact that different local instances of an (initially identical) cgMLST scheme quickly diverge from each other, as new allele sequences are added, leading to the assignment of the same allele number for different allele sequences, or of the same allele sequence to be attributed to two or more different allele numbers. This can only be prevented by real-time synchronization of the local allele database with a (centrally) curated cgMLST allele nomenclature server, as implemented in Ridom SeqSphere and chewieNS (Mamede et al., 2020).

One possible solution is the replacement of chronologically numbered allele numbers with numbers or strings that are directly related to the underlying allele sequence. Using the allele sequence itself is impractical, as it hugely increases the size of an allele profile. Alternatively, *allele hashes* can be derived directly and unambiguously from the allele sequence. Allele hashes uniquely map the nucleotide sequence into a fixed-size hash value. Thus, independently discovered, identical allele sequences always result in the identical allele hash. This has the major advantage of allowing a decentralized nomenclature-free allocation of sequencing types, with no need for harmonization with a central unit. The practicability of the hashing approach has been demonstrated by the bioinformatic tool SISTR (Yoshida et al., 2016) for the purpose of serotyping *Salmonella* spp. and for *Clostridium difficile* in (Eyre et al., 2019).

Existing bioinformatic cgMLST standalone tools differ not only in their implemented cgMLST scheme but also in their specific allele calling algorithm and whether the source code is freely available, i.e., open- or closed-source. Two popular commercial solutions (source code not freely available) with a graphical user interface (GUI), which can be run on Windows systems, are Ridom SeqSphere+^8^ and Bionumerics^9^. Both employ a closed system with a central nomenclature server. In comparison, two open-source, command-line tools, MentaLiST (Feijao et al., 2018) and chewBACCA (Silva et al., 2018), allow users to utilize their own cgMLST scheme. ChewBBACA is a comprehensive pipeline for cgMLST calling, as well as for the creation of new cgMLST schemes. The allele calling algorithm of chewBACCA considers the Blast Score Ratio in order to determine the allele sequences. Among the advantages of chewBBACA is that it automatically incorporates novel alleles into a scheme. Its usability has furthermore been demonstrated in numerous studies (Macedo et al., 2019; Lüth et al., 2020; Pinto et al., 2020; Uelze et al., 2020a).

Here, we present *chewieSnake*, an automated analysis pipeline that encompasses the whole analysis process from reads or draft assemblies to a final user-friendly cgMLST report. The pipeline implements chewBBACA for allele calling and performs allele hashing, computation of an allele distance (AD) matrix, and a minimum-spanning tree, as well as a clustering analysis by AD. The analysis results are summarized in an interactive HTML report.

We demonstrate the workflow’s usability by analyzing a large public database for *Salmonella enterica* and validate it by comparing it to two publically available, state-of-the-art methods—Enterobase and NCBI pathogen detection. Additionally, we simulate a scenario of decentralized allele calling and central cluster analysis.

Finally, we want to emphasize that the presented method is not restricted to *Salmonella* spp. but is readily applicable to all bacterial taxa, provided a suitable cgMLST scheme is available.

## Methods

### Implementation and Availability

The provided software consists of two workflows—*chewieSnake* and *chewieSnake_join*. Additionally, several modules of the software are available on their own and can be applied in conjunction with other analysis workflows. The software is available open-source^10^ and can be easily installed using bioconda (Grüning et al., 2018) or docker (Merkel, 2014).

#### *ChewieSnake* Workflow

**FIGURE 1:**
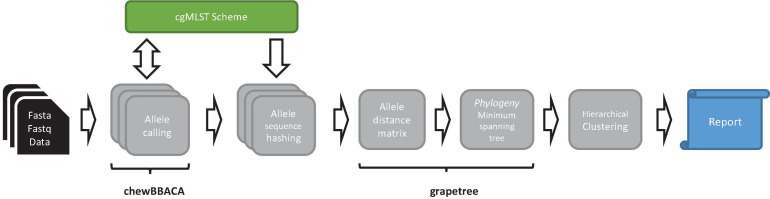
Schematic representation of the *chewieSnake* workflow. The workflow starts from a set of assemblies (fasta data) or a set of raw reads (fastq data), which are then assembled into fasta files. On these, cgMLST allele calling is performed using the tool chewBBACA, utilizing a designated cgMLST scheme, to which newly found alleles are added in the process. The resulting allele profiles are converted to hashed allele profiles. All profiles are subsequently combined; and an allele distance matrix and a minimum-spanning tree phylogeny are computed with the tool GrapeTree. Then, cluster types are generated by hierarchical clustering at different allele distance thresholds. Finally, the results are summarized in an interactive HTML report.

*ChewieSnake* implements a workflow for allele calling, computation of the ADs, and a minimum-spanning tree, with analysis results summarized in an interactive HTML report. Central to the workflow is the concept of allele hashes that allow a nomenclature free comparison of allele profiles (see Figure 1).

##### Snakemake workflow

The workflow consists of a Snakemake pipeline (Koster and Rahmann, 2012) with a set of assemblies (or sequencing reads) as input listed in a sample sheet. Snakemake resolves all dependencies in a directed acyclic graph (DAG), runs all necessary components for each sample, merges all results, and renders the final HTML report using Rmarkdown (rmarkdown, 2020) (see also Supplementary Figure 1). The design of the workflow is such that samples may be added continuously without the need to re-compute analysis results for previously analyzed samples, thus optimizing the computational time/resources for maintaining an allele profile database containing a large number of samples. Nevertheless, the clustering, phylogeny, and report are updated whenever new samples are provided.

##### Allele calling

The basis for the central allele calling step is the assembly based allele caller chewBBACA (Silva et al., 2018). Its main concept lies in the identification of coding sequences (CDSs) using prodigal (Hyatt et al., 2010). While existing alleles are detected via exact sequence matching, novel alleles are quality controlled via a BLASTP routine. For the usage of chewBBACA in a Snakemake workflow—and in particular to allow the continuous addition of samples—a fixed version (2.12.0) was included in the *chewieSnake* repository with minor adaptations to their output: chewBBACA is called for one sample at a time, and the output is stored in a folder with the sample’s name. Possibly conflicting parallel writing to the allele database is blocked.

##### Allele hashing

The allele profiles as provided from chewBBACA are reformatted into a GrapeTree compatible format; and for each sample, the allele numbers—as provided in the applied cgMLST scheme—are converted into allele hashes. This step guarantees that the same allele hash is assigned for any identical allele sequence. Thus, independent identification of alleles for different samples and on different computing facilities always leads to the same allele hash and therefore clustering result.

The provided function *alleleprofile_hasher.py* looks up the allele sequence for each allele number in an allele profile in the scheme and computes a CRC32 hash using python’s zlib package, yielding a unique integer.

The function *alleleprofile_hasher.py* can be used as a standalone program, e.g., if a user prefers to use their own analysis workflow (based on chewBBACA) and thus allows the nomenclature free sharing of allele profiles regardless of the presented *chewieSnake* workflow.

##### Unique hashed sequence types

To generate unique sequence types for each sample (hashIDs), the hashing approach is also applied. For each allele profile, the allele hashes (including possibly missing loci) are combined into a single string, and a hash value of the string provides the unique sequence type. It should be noted that this approach is stricter than clustering samples at 0 AD, since missing alleles are explicitly accounted for.

The provided function *hashID.py* can be used standalone, thus enabling users to infer hashed sequence type for any kind of allele profile.

##### Allele distance matrix and minimum-spanning tree

Based on the allele profiles, the workflow uses GrapeTree (Zhou et al., 2018) for the computation of the AD matrix. GrapeTree allows the computation of the distance in various ways, which differ in how missing alleles are treated. In addition, GrapeTree is also used to infer the phylogeny of all samples with minimum-spanning trees. The resulting Newick file can be readily rendered with GrapeTree’s graphic visualization software or other phylogenetic visualization tools. This step and all subsequent steps are repeated whenever new sample data are added to the workflow.

##### Sample clustering

*ChewieSnake* hierarchically clusters the distance matrix of all samples (using, e.g., single linkage or average linkage hierarchical clustering) using the R-function *hclust* (R Core Team, 2013). Subsequently, given the clustering, a set of pre-defined thresholds assign cluster numbers to all samples at different thresholds—using dendextend (Galili, 2015). This provides so-called *cluster addresses* or *cluster zip-codes*, which allow an additional description of the relatedness of a set of samples. This is a similar approach to SNP addressed in SnapperDB (Dallman et al., 2018) or HierCC in Enterobase (Zhou et al., 2020b).

The function *Clustering_DistanceMatrix.R* is provided in the scripts directory and can be used standalone for any kind of distance matrix, including SNP distance matrices.

##### HTML report

After processing and collection of all sample data, the entire dataset is summarized in an interactive user-friendly HTML report. The report is organized into different tabs and designed with the aim to give all scientists (regardless of bioinformatic background) access to the most important results:

In the *Allele statistics* tab, key quality assessment parameters of the allele calling step are shown for each sample, such as the number of alleles found—as well as the reason for missing alleles. The *Allele distance table* tab features a searchable table with all pairwise ADs. The *Allele distance matrix* tab provides a colored and zoomable visualization of the distance matrix. The *Clustering* tab presents a searchable table of the clustering address at the pre-defined thresholds. Furthermore, single-linkage trees are printed for all identified clusters at the pre-defined cluster threshold. The *Minimum-spanning tree* tab displays a simple (static) visualization of the phylogenetic tree. Lastly, the tabs *Links to files*, *Config and parameters*, and *Help* give further directions for more detailed analysis and enable optimal reproducibility.

An example report for a small test set can be found at https://bfr_bioinformatics.gitlab.io/chewieSnake/report_chewiesnake.html.

##### User input and parameter choice

The user has control over a large set of parameters, which are available with the command *chewieSnake.py–help*.

Importantly, if only reads are available for analysis, *chewieSnake* can also be given a set of reads as input, which it then trims using fastp (Chen et al., 2018) and subsequently assembles into draft genomes using shovill^11^, before proceeding with the Snakemake workflow. Further important parameter choices are the GrapeTree distance method (see above), the clustering method, and thresholds, as well as chewBBACA specific parameters. All parameters are transparently saved and included in the report for reproducibility.

##### Possibilities in *chewieSnake* for inter-lab –comparison

The *chewieSnake* workflow contains the –comparison option to compare a set of query data with a pre-computed allele profile. This allows the identification of clusters between query data and comparison data. The workflow processes the query data in the same fashion as described above. When the (hashed) allele profiles are computed, these are joined with the comparison allele profiles. Next, all samples from the comparison allele profiles that match to any of the query data within a predefined *joining_threshold* are extracted; and a distance matrix, distance table (query vs. comparison data), and a minimum-spanning tree are computed. Again, all findings are summarized in an HTML report, highlighting the matches between the two datasets. In conclusion, this approach allows a quick screening of a potentially large comparison allele database for matches to queries of interest, e.g., in an outbreak situation. This strategy might also be useful when a comparison of newly sequenced data to existing allele data is desired.

An example report is provided at https://bfr_bioinformatics.gitlab.io/chewieSnake/report_chewiesnake_comparison.html.

#### *ChewieSnake_Join* Workflow

**FIGURE 2:**
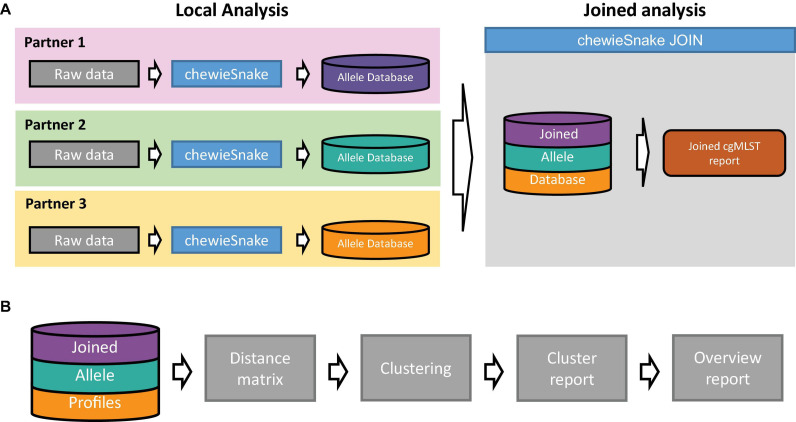
Schematic representation of the c*hewieSnake_join* workflow. **(A)** A set of laboratories locally employ the *chewieSnake* workflow to process incoming raw sequencing data. The resulting allele profiles are stored in individual allele databases, which are subsequently joined with the *chewieSnake_join* workflow. In addition, the *chewieSnake_join* workflow computes a joined cgMLST report. **(B)** Detailed description of the *chewieSnake_join* workflow. From the joined allele profiles, the allele distance matrix is computed, followed by cluster typing of the entire dataset. Detailed reports for each cluster as well as an overall report summarize the results in an interactive HTML format.

The central allele hashing concept within the *chewieSnake* workflow allows the nomenclature-free comparison of allele profiles generated by different laboratories. To facilitate the comparison and establish an inter-laboratory outbreak clustering nomenclature, the *chewieSnake_join* workflow conducts all necessary steps from individual *chewieSnake* results to a joined clustering report. The conceptual model is depicted in Figure 2A, whereas the essential steps are shown in Figure 2B. In the following, we describe the core elements of the workflow in more detail.

##### Compiling pre-computed allele data from a set of laboratories

All laboratories setup and run the *chewieSnake* workflow independently on their set of data. Laboratories can validate their results by inspecting the allele quality and the local clustering results.

The central results (provided in the files allele_profiles.tsv, allele_statistics.tsv, timestamps.tsv) need to be collected from each laboratory. The location of all files is provided in an *allele sheet* (a tsv file containing each laboratories’ acronym, as well as paths to the allele profiles, allele statistics, and timestamps). This is the input to the *chewieSnake_join* workflow.

The contributed data can be updated continuously. On each execution of *chewieSnake_join*, the workflow identifies new samples and repeats subsequent steps automatically where necessary.

##### Joining allele and associated data

The individual allele profiles (and other files) are joined into single files, and a file tying the laboratory of origin to the sample name is generated. Subsequently, in analogy to the *chewieSnake* workflow, a distance matrix and minimum-spanning tree are computed on the basis of the full dataset.

##### Cluster and subcluster identification

The joined AD matrix is input to the clustering module of *chewieSnake_join*. Again, hierarchical clustering together with a predefined threshold divides the dataset into individual clusters (and unrelated orphan samples). Since the clustering may assign new cluster numbers each time it is invoked, special emphasis is put on a stable clustering nomenclature: The cluster numbers are matched to a list of cluster names (which can be numbers such as *CT_0001* or predefined names such as *Cluster_Kairo*), whenever the clustering is repeated, e.g., after addition of more samples. Hereby, it is guaranteed that the same cluster name is assigned to the same set of samples. Exceptions such as the merging of two or more clusters are considered and handled adequately. In a similar fashion, for the samples in each cluster, a subclustering at a lower subclustering threshold is performed—again using stable names such as alpha and beta—thus allowing an intercommunicable cluster nomenclature at a finer level.

##### Additional options and parameters

Apart from setting clustering methods and thresholds, a number of additional options can be invoked in *chewieSnake_join*.

In particular, individual representative samples can be matched to external cluster names (using the *–external_cluster_names* flag). For instance, the latter can describe epidemiologically defined clusters—independent from the molecular data. *ChewieSnake_join* links these external cluster names to the internal cluster names, which may aid the tracking of established clusters in the report (see below). Additionally, samples can be assigned to e.g., serovars or larger clades using the *–serovar_info* flag. Again, this might facilitate the cluster analysis provided in the report. The matching of these additional data proceeds by providing a list of representative samples and their association to external clusters and serovar, respectively. *ChewieSnake_join* associates these representative samples to the identified clusters, and this association becomes accessible in the report.

The pool of names for the cluster naming can be modified by providing a list of available cluster names with the *–cluster_names* flag.

All available options and parameters can be inspected using *chewieSnake_join.py–help*.

##### Joined HTML report

Central to the facilitation of a joined outbreak analysis is the *chewiesnake_join* HTML report. This report summarizes all findings from the data aggregation and clustering. It is organized into different tabs, as follows.

The *Overview tab* condenses the information about samples per laboratories, date of sample analysis, and number of clusters shared between laboratories. The *Cluster summary* tab provides the central information for the clustering analysis in a first (searchable) table that is organized by cluster. It provides readily accessible information such as cluster size, latest updates, duration (age) of cluster, cluster nomenclature, and the matching to external cluster names and serovar. Another table lists all samples and their respective cluster assignments. The *Orphans* tab displays all samples that, under the chosen threshold, do not belong to any existing cluster, together with their distance to the closest cluster. The *Inter-cluster relation* tab describes the more global relationship between clusters. The *Allele QC* tab allows an inspection of the allele quality for the entire dataset. The tabs *Links to files*, *Config and parameters*, and *Help* provide additional information.

An example report is provided at https://bfr_bioinformatics.gitlab.io/chewieSnake/report_chewiesnake_join.html.

For each identified cluster, a more detailed cluster report is available. This report summarizes the subclusters, the AD matrix of all samples in the cluster, the time evolution of the cluster (according to the timestamps of the analyses), and the cluster’s and all subcluster’s phylogeny.

An example report is provided at https://bfr_bioinformatics.gitlab.io/chewieSnake/clustering/CT_0004/clusterreport.html.

### Dataset for Evaluation

#### Raw Data

In total, 1,263 WGS sequencing data were obtained from BioProject PRJEB31846. The dataset comprises diverse *S. enterica* serovars collected between the years 1999 and 2019 and sequenced by the National Reference Laboratory for *Salmonella* using the Nextera XT or DNA Flex kit (Illumina GmbH, München, Germany) on Illumina MiSeq and NextSeq instruments. The data are described in more detail in (Uelze et al., 2019).

#### Assembly

Data were trimmed [with fastp (Chen et al., 2018), version 0.19.5] and assembled [with shovill-spades (see text footnote 11), version 1.1.0] using the AQUAMIS pipeline^12^ (version v1.2.0) (Deneke et al., 2021). All samples passed basic quality checks, such as sufficient base quality, coverage depth, genome length, and contig number. Furthermore, no evidence for sample contamination was detected. The assemblies are available under https://zenodo.org/record/4338293.

#### cgMLST Scheme

The cgMLST scheme for *Salmonella* was downloaded on June 11, 2018, from Enterobase (Zhou et al., 2020a) using the web API. As the sequencing data were published at a later date, this ensured that the downloaded cgMLST scheme did not already contain allele numbers from our dataset. Only this strategy allows an unbiased analysis of the allele calling process and in particular the assignment/identification of novel alleles, as would be the case for newly generated sequencing data unknown to a specific reference allele database. The scheme is available under https:// zenodo.org/record/4724927.

#### Allele Calling

The assembled draft genomes were analyzed using the *chewieSnake* workflow. All parameters and software versions are specified in the Supplementary Data Sheet 2. The resulting *chewieSnake* report is available under https://bfr_bioinformatics.gitlab.io/chewiesnake_publicationdata/chewiesnake/cgmlst_report.html.

#### Simulation of Decentralized Analysis

The dataset was split randomly into three non-overlapping sub-datasets. The membership of each sample is listed within Supplementary Data Sheet 1. A cgMLST analysis with *chewieSnake* (with the same software version and parameters as for the full dataset) was performed separately on each sub-dataset. For each analysis, an identical but separate allele database was employed.

In order to simulate a decentralized surveillance system over a given time span, the timestamp files—which originally contain the date of the analyses—were modified to random dates from the period of January 2020 to October 2020. Other than for visualization purposes, this had no impact on the analyses.

Results were joined using the *chewieSnake* joining workflow (see above). All parameters are specified in the Supplementary Data Sheet 2. The resulting joined report is available under https://bfr_bioinformatics.gitlab.io/chewiesnake_publicationdata/chewiesnake_join/report.html.

#### Enterobase Data

Available cgMLST metadata for Bioproject PRJEB31846 were searched and downloaded from https://enterobase.warwick.ac.uk/species/index/senterica on November 2, 2020. The set of allele profiles was downloaded on November 2, 2020, and subsequently filtered for all entries of BioProject PRJEB31846. A total of 1,158 data were also found on Enterobase.

An AD matrix was computed from the allele profiles using GrapeTree in complete analogy to the *chewieSnake* workflow. Subsequently, the distance matrix was hierarchically clustered, and a cluster address was assigned using the provided script *Clustering_DistanceMatrix.R.* Thus, all analysis steps from the allele profiles were identical to the *chewieSnake* workflow.

#### National Center for Biotechnology Information Data

*Salmonella* SNP distances were obtained from NCBI Pathogen detection^13^ on September 10, 2020, corresponding to version PDG000000002.1968. With the use of a custom script, the pairwise SNP distances were filtered such that all distances of pairs originating from Biosamples associated with BioProject PRJEB31846 were retained. Overall, 811 samples were part of an SNP cluster on NCBI pathogen, and the samples were found in 120 distinct SNP clusters. Note that the definition of an SNP cluster on NCBI pathogen does not correspond to the hierarchical clustering studied in this contribution but rather to the set of samples with the same reference for SNP calling.

For this analysis, the SNP distance data were hierarchically clustered, and cluster numbers were assigned to each sample using a custom script in the same fashion as described above.

### Methods for Comparison Analysis

#### Comparison of Pairwise Distances

**FIGURE 3:**
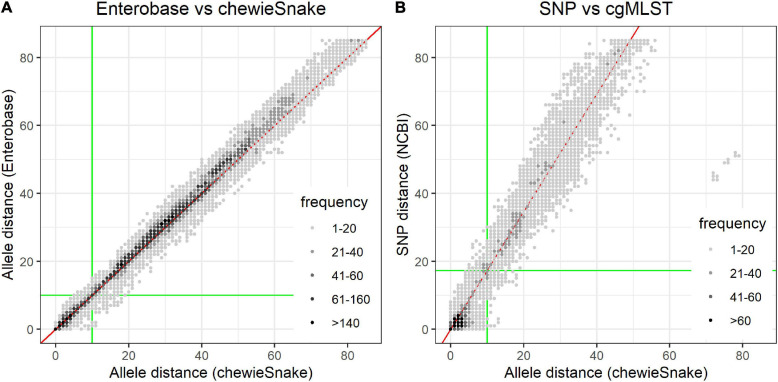
Pairwise distances show very high method congruence. **(A)** Comparison of allele distance between Enterobase and *chewieSnake*. **(B)** Comparison of NCBI_pathogen SNP and *chewieSnake* allele distance. The gray shading indicates the frequency of observed pairwise distances. Both figures show the very high concordance between the different methods. This is also reflected in the Spearman correlation coefficients (0.984 and 0.973). The slope of a linear fit (red line) to the NCBI_SNP and *chewieSnake* comparison is ∼1.73; i.e., on average, 1 allele difference corresponds to 1.73 single-nucleotide polymorphisms. For the computation of sensitivity and specificity, the green lines mark clustering thresholds. All points in the lower left quadrant are counted as true positives, and all points in the upper right quadrant as true negatives, whereas the points in the top left and bottom right quadrants are false positives and false negatives, respectively. Using these thresholds also leads to the high adjusted Wallace and adjusted Rand coefficients (see Tables 1, 2). Though overall the agreement is very high, some remaining differences at low distances may still influence interpretation of the very fine-grained phylogeny. Note that for the Enterobase comparison, only distances below 85 AD were displayed.

Pairwise distances of each method were obtained as described in the individual sections above. Each resulting distance matrix was read into R and converted to a linearized table with the pair’s sample names as keys. The linearized distance tables resulting from different methods were matched using the keys, followed by redundancy and self-hits removal. Based on these distance-method-comparison tables (provided in the Supplementary Data sheet 1), the Spearman correlation was computed, and the pairwise distance comparison plots were drawn (Figures 3A,B).

#### Computation of Sensitivity/Specificity Values

For each method combination, the pairwise distances of all sample pairs were obtained and a distance threshold was chosen, such that samples could be grouped into possible related clusters. Though no single fixed threshold is sufficient and advisable for the differentiation of outbreak to non-outbreak strains (Simon et al., 2018; Radomski et al., 2019), an allele threshold of 10 is considered suitable for an initial clustering (Besser et al., 2019; Uelze et al., 2021). For comparability with cgMLST clustering results, an SNP threshold of 17 was chosen (see section “Comparison With National Center for Biotechnology Information Pathogen Single-Nucleotide Polymorphism Results” for details), which is in agreement with Pightling et al. (2018). If the pairwise distance was below or above the threshold for both methods under comparison, it was considered as true positive (TP) or true negative (TN), respectively. If the pairwise distance was below the threshold for only one of the methods under comparison, it was considered as false positives (FPs) (method 1) or false negatives (FNs) (method 2) (see also Figure 3). From the TP, TN, FN, and FP values, the corresponding values for specificity and sensitivity were derived.

#### Comparison of Clustering

The distance matrices resulting from each method were hierarchically clustered and divided into clusters addresses using the thresholds described above. Direction-dependent concordance of clustering, expressed by the adjusted Wallace coefficient, was calculated with the Comparing Partitions online tool available at http://www.comparingpartitions.info (Carriço et al., 2006). The adjusted Wallace coefficient allows the direct interpretation of clustering results, ensuring that an agreement is not caused by chance (Pinto et al., 2008; Severiano et al., 2011). The same resource also enables the computation of the adjusted Rand index, which allows the estimation of the global congruence of two typing approaches (Hubert and Arabie, 1985).

## Results

### *ChewieSnake* Workflow

We developed *chewieSnake*, an end-to-end analysis workflow for allele calling, allele profile clustering, and computation of a minimum-spanning tree (see Figure 1). Its core components rely on a number of state-of-the-art bioinformatic tools including chewBBACA and GrapeTree. Central to the workflow is the concept of allele hashes that allow a nomenclature-free comparison of allele profiles.

From the user perspective, *chewieSnake* provides an intuitive and simple-to-use workflow that orchestrates all necessary steps from a set of reads or assemblies to a highly informative, interactive cgMLST report displaying sample relatedness and their phylogeny.

Thus, *chewieSnake* enables scientists without profound bioinformatic background to perform a set of complex tasks for a reproducible high-throughput analysis that can be readily applied in outbreak investigations.

Moreover, *chewieSnake*’s built-in hashing algorithm includes the possibility to directly compare allele data generated independently, for instance, on different sites. In particular, the *chewieSnake_join* workflow provides a straightforward and easy-to-implement method for a distributed outbreak investigation system. The workflow provides users with highly informative HTML reports, which allow the quick identification of shared clusters between laboratories, the definition of a common cluster nomenclature, and detailed reports for each identified cluster (see Figure 2).

The *chewieSnake* workflow and program codes are fully open-source and can be obtained from https://gitlab.com/bfr_bioinformatics/chewieSnake. Details on the workflow components are given in section “Implementation and Availability.”

### Workflow Validation

#### Summary of *ChewieSnake* Analysis

The dataset contains WGS data of 1,263 sequenced *S. enterica* isolates from BioProject PRJEB31846. All samples could be analyzed with *chewieSnake*, and sufficient loci for cgMLST analysis were found. On average, 97.8% of the loci were found, and an allele sequence could be identified. More than 95% of all loci were found for all but two samples. The sample with the least fraction featured 94.3% of all loci, corresponding to 170 missing loci. Therefore, sufficient targets/loci for cgMLST analysis could be found for all samples.

Overall, we detected 33,387 novel alleles not previously known to the cgMLST scheme. For each sample, we found 26.4 novel alleles on average, with only 17% of the samples containing no novel allele. Furthermore, novel alleles were evenly found at the beginning and end of the experiment, showing no signs of saturation (see Supplementary Figure 3). Thus, finding novel alleles is very common, and properly treating these alleles is key for a comparable data analysis (see also Supplementary Figure 4).

For a distance cutoff of 10, we found that the data separate into 170 different clusters containing at least two samples. A total of 439 samples did not cluster within the threshold (orphan samples) (see also Supplementary Figure 2).

#### Repeatability of Analysis

We repeated the entire analysis using the original, unaltered Enterobase scheme, i.e., by running *chewieSnake* on the same dataset again. Overall, we found large repeatability. The same novel alleles were identified in both datasets and assigned the identical allele hashes. Thus, an identical allele sequence consistently leads to an identical allele hash. Moreover, the same number of clusters was detected.

However, in some cases, minor allele differences were found. Among the epidemiologically relevant sample pairs with distances below 20 AD, 2% of the sample pairs differed by 1 AD. The reason for these differences lies in the different order of execution of the sample allele calling (which is not pre-determined by Snakemake but randomly assigned on each execution) and the treatment of novel alleles by chewBBACA. The strict BLASTP step in chewBBACA might discard novel alleles, while the allele might be counted as an exact match if the allele sequence is already known to the database. If a sample featured a missing locus in the first analysis, but in the second analysis an allele difference was found on this locus, the allele difference would be reduced by 1 in the first analysis compared with the second. Indeed, 720 and 562 loci were missing in only one of the analyses, but not both.

The Supplementary File 1 provides a more detailed analysis of the dataset. The analysis of the dataset is summarized in the cgmlst report https://bfr_bioinformatics.gitlab.io/chewiesnake_publicationdata/chewiesnake/cgmlst_report.html.

#### Comparison With Enterobase

The comparison between all sample combinations reveals a very high concordance between Enterobase and *chewieSnake* ADs with a Spearman correlation of 0.984 (see Figure 3A). Most distances are found at or near a line with slope 1 (a linear regression reveals a slope of 1.02). This extends also to high ADs (see Supplementary Figure 5). A histogram of the method differences of epidemiologically relevant sample pairs (i.e., within 30 AD) reveals that distances mostly differ by one or two alleles only, with a mean of non-zero method differences of 1.8 AD. We also found a tendency of higher distances in Enterobase (see also Supplementary Figure 6).

When performing a threshold analysis at 10 AD, sample pairs are found with 0.96 sensitivity and 0.99 specificity within or outside the same distance threshold (amounting to an accuracy of 0.99). Moreover, a closer look at the FPs and FNs reveals that the misclassified samples differ by a few (mostly one or two) allele differences only. Thus, these apparent misclassifications result from the assumption of a fixed threshold. The maximum AD in Enterobase for a sample pair within 10 AD in *chewieSnake* cluster was 15, and the average of all putatively misclassified pairs in Enterobase was 11.8.

When hierarchically clustering the distance matrix obtained by both methods, the (direction-dependent) adjusted Wallace coefficient was 0.955 and 0.975 (see Table 1). The adjusted Rand index also reveals a high method congruence (see Table 2).

**TABLE 1:** (Direction-dependent) adjusted Wallace coefficient for all method comparisons.

	*chewieSnake*	*chewieSnake_join*	Enterobase	NCBI pathogen
*chewieSnake*	1	0.992	0.955	0.922
*chewieSnake_join*	1.000	1	0.964	0.931
Enterobase	0.975	0.975	1	0.935
NCBI pathogen	0.968	0.968	0.964	1

*For each method, the distance matrix was hierarchically clustered using single-linkage trees. The clustering is based on a cluster threshold of 10 for the cgMLST methods and of 17 for the SNP method (NCBI). SNP, single-nucleotide polymorphism; NCBI, National Center for Biotechnology Information.*

**TABLE 2:** Discriminatory power according to adjusted Rand index.

	*chewieSnake*	*chewieSnake_join*	Enterobase	NCBI pathogen
*chewieSnake*				
*chewieSnake_join*	0.996			
Enterobase	0.965	0.969		
NCBI pathogen	0.944	0.949	0.949	

*All methods show very high discriminatory power, above >0.94. Noteworthy is that *chewieSnake* and *chewieSnake_join* have a nearly perfect accordance. NCBI, National Center for Biotechnology Information.*

It is important to note that very similar conclusions can be drawn from different allele thresholds. Evidently, method differences by one or two alleles have a larger impact on clusters determined at lower compared with larger distance thresholds. Thus, a very low threshold may lead to a decrease in the cluster congruence. However, fixed thresholds are most relevant for linking samples to outbreak clusters, while for a closer analysis of the phylogeny, the exact tree topology is more useful.

#### Comparison With National Center for Biotechnology Information Pathogen Single-Nucleotide Polymorphism Results

Overall, there is a linear relationship between SNP and AD (see Figure 3B). A linear model fit reveals that statistically 1 AD corresponds to 1.73 SNP differences. The Spearman correlation reads 0.973. This suggests that samples within an AD of 10 should have an SNP distance of smaller or equal to 17 (definition of TPs). A threshold analysis shows a concordant clustering decision of 95% between cgMLST and SNP analysis (sensitivity—all points in the lower left quadrant in Figure 3B). Similarly, both methods agree on assigning a sample pair in 96% of all cases not to be part of a cluster (see Figure 3B).

We hierarchically clustered the distance matrices from both methods (cgMLST and SNP.) The resulting (direction-dependent) adjusted Wallace coefficient was 0.922 and 0.968, respectively (see Table 1). In addition, the adjusted Rand index reveals a high method congruence (see Table 2). Again, similar conclusions can also be drawn from different allele and SNP thresholds.

At small distances, i.e., well below the applied threshold, SNP and ADs are more scattered than between the cgMLST approaches. Thus, although samples can be concordantly associated with outbreak clusters, the detailed phylogeny may differ. This is, however, not surprising given the very different nature of SNP and cgMLST approaches.

Table 1 also yields the congruence between the reference methods Enterobase and NCBI pathogen. It shows that the adjusted Wallace coefficient between *chewieSnake* and the reference methods was even higher than the congruence among the reference methods.

#### Simulated Decentralized cgMLST Analysis

The concordance between the *chewieSnake* analysis on the entire dataset (centralized approach, see above) and a simulated decentralized *chewieSnake_join* analysis has been evaluated. For the latter, the original dataset was randomly divided into three equally sized, non-overlapping subsets, analyzed individually with *chewieSnake* and joined subsequently with *chewieSnake_join* (see section “Methods”).

The ADs obtained from centrally and decentrally analyzed samples are nearly identical—the Spearman correlation is 1 (0.9999997). Table 3 shows that at almost all thresholds with the same number of clusters were identified (the only exception was 160 vs. 159 distinct clusters at 5 AD). Furthermore, almost all sample pairs fall into the same 10 AD cluster, amounting to over 99.9% sensitivity and specificity. In the three exceptions, the sample pairs had 10 AD and 11 AD, respectively, not leading to a different epidemiological interpretation. The (direction-dependent) adjusted Wallace coefficient was 0.992 and 1.000, respectively (see Table 1). The adjusted Rand index reads 0.996 as well, demonstrating that centralized and decentralized allele calling is fully compatible (see Table 2).

**TABLE 3:** Cluster counts for *chewieSnake* and simulated *chewieSnake_join* analysis.

	1,000	200	100	50	20	10	5	1
*chewieSnake*	50	71	83	97	150	170	159	102
*chewieSnake_join*	50	71	83	97	150	170	160	102

*The number of identified clusters agrees at all different thresholds with a single difference for CT_5.*

The origin of the detected small differences was found to be the same, as discussed in the repeatability analysis of the *chewieSnake* analysis (see above). Importantly, the allele calling always leads to the same allele sequence and hence the same allele hash. Instead, the differences are due to the different execution order of the samples’ allele calling, which impacts the discovery of novel alleles and exclusion of loci in chewBBACA. Potential allele differences between a sample pair may be masked when in one sample the locus was classified for instance as a *non-informative paralogous hit*. This classification is, however, subject to whether the allele was already known to the scheme prior to the allele calling. For the present dataset, 246 loci in samples in the centralized dataset and 191 loci in samples in the decentralized dataset were discarded as paralogues. Additionally, eight loci were discarded as being too close to a contig border. Hence, subtle but epidemiologically negligible differences may occur due to the different order of samples during the allele calling step of chewBBACA.

## Discussion

In this study, we demonstrated the broad utility and validity of our newly developed end-to-end cgMLST analysis workflow. The method successfully determines clusters fully unsupervised without the need of user intervention. The proposed approach works both for a centralized data analysis, as well for a decentralized analysis followed by central clustering. This is a unique feature of the implemented allele hashing algorithm. Thus, the research community benefits from a versatile, easy-to-use tool that can be readily applied for molecular surveillance. It shall be noted that the presented method is readily applicable to all taxa where a suitable cgMLST scheme is available.

In comparison with existing approaches, for example, Ridom SeqSphere+ (with cgMLST.org) or the very recent contribution chewieNS (Mamede et al., 2020), *chewieSnake* does not rely on a central allele nomenclature. In the case of chewieNS, chewBBACA is also implemented as the underlying allele calling software and thus is prone to the same distinction between exactly determined and newly inferred alleles. The chewieNS approach functions without an allele hashing step on the user side, but as a downside, it introduces the requirement to synchronize the scheme prior to every allele calling. The synchronization is critical for ensuring comparable allele numbers and might be challenging in outbreak situations, when suspected samples may be sequenced and analyzed at different sites at the same time. Nonetheless, if a nomenclature-based solution has been used to create allele profiles at different sites, the *chewieSnake_join* workflow can be used for merging of the allele results and a joint cluster interpretation. Also, the concept of allele hashing could be directly incorporated in the allele calling software, e.g., in future upgrades of chewBBACA.

As pointed out above, the discovered remaining differences between central and decentralized approaches are not related to the hashing algorithm but rather to unique features of the underlying chewBBACA software. These issues could therefore only be remedied by substantial changes to the chewBBACA allele calling algorithms. However, as discussed previously, the effect on cluster detection is negligible.

Results of *chewieSnake* are in strong agreement with Enterobase, although both approaches differ substantially regarding their implementation. This might be somewhat expected given that both methods rely on the same cgMLST scheme. Our analyses revealed that distances in Enterobase tend to be larger, which are mainly caused by the greater number of loci available in Enterobase. Due to the CDS prediction step in chewBBACA, fewer loci are accessible in *chewieSnake*, leading to a smaller effective scheme size in the latter.

The accuracy for correct cluster detection was furthermore confirmed by comparison with an independent complementary method—SNP calling from NCBI pathogen detection. Overall, we found a very high consistency. As anticipated, ADs and SNP distances are not fully comparable. On the one hand, an allele pair might differ by more than one SNP—thus, the AD underestimates the SNP distance. On the other hand, allele differences might originate from other mutation events such as indels (insertions and deletions) and are therefore not characterized as an SNP. In the latter, allele difference might also overestimate the SNP distance. Nonetheless, the large concordance between SNP and cgMLST results also boosts confidence for the epidemiological assessment.

Alternatives to self-hosted and open-source cgMLST analysis workflows such as *chewieSnake* are central analysis systems such as Enterobase (Zhou et al., 2020a) (or also NCBI pathogen detection (see text footnote 1), INNUENDO (Llarena et al., 2018), etc.]. Among the advantages are greater standardization, more rigid control of parameters, and a larger data pool [e.g., NCBI pathogen and Enterobase incorporate all data from NCBI sequence read archive (SRA)]. Conversely, these approaches limit flexibility in terms of changing parameters and selecting a scheme and a species of choice. Also, *chewieSnake* and in particular its decentralized feature might render it more easy to share data and results when data confidentiality concerns apply. Furthermore, given that the amount of data present in public repositories (and consequently their computational needs) grow exponentially, *chewieSnake* is our approach to envision future tractability in terms of lean computing and storage resources by decentralizing and outsourcing the core analysis steps to the data providers.

In addition to the presented novel methods, the detailed analysis and contributed data provide an excellent dataset for the validation of further methods (and parameters/schemes). For this aim, one simply needs to compute a distance matrix and either compare the pairwise distances or cluster the distance matrix to a desired threshold for the imputation of relevant metrics such as the adjusted Wallace coefficient (see a more detailed sketch in the Supplementary Data sheet 2 and the provided scripts). It is worth noting that the validation approach of this contribution is also in agreement with the forthcoming ISO 23418 titled “Whole genome sequencing for typing and genomic characterization of foodborne bacteria—General requirements and guidance (ISO/DIS 23418, 2020)” for validation of bioinformatic software, in particular by validating with publicly accessible data and methods. However, the present analysis also indicates that further harmonization of bioinformatic approaches is needed (Jagadeesan et al., 2019; Coipan et al., 2020). In the cases where this is not possible (e.g., cgMLST vs. SNP), the expected differences in the epidemiological interpretation from different methods need to be further discussed within the research community.

## Data Availability Statement

The developed software can be found in https://gitlab.com/bfr_bioinformatics/chewieSnake, as well as in bioconda and Docker Hub (https://hub.docker.com/r/bfrbioinformatics/chewiesnake). The raw data analyzed for this study can be found in BioProject PRJEB31846. The assembled genome data can be found under https://zenodo.org/record/4338293 and the used cgMLST scheme under https://zenodo.org/record/4724927. The workflows’ analyses can be found in https://bfr_bioinformatics.gitlab.io/chewiesnake_publicationdata. All further relevant data for reproducing the results can be found in the Supplementary Data Sheets 1, 2.

## Author Contributions

CD, LU, ST, and BM designed the project. CD wrote the software with support from HB and ST. CD, LU, and ST conducted the analysis. All authors wrote and approved the manuscript.

## Conflict of Interest

The authors declare that the research was conducted in the absence of any commercial or financial relationships that could be construed as a potential conflict of interest.

## Footnotes

1 https://www.ncbi.nlm.nih.gov/pathogens/

2 https://achtman-lab.github.io/GrapeTree/MSTree_holder.html

3 https://online.phyloviz.net/index

4 https://enterobase.warwick.ac.uk/

5 https://bigsdb.pasteur.fr/

6 https://www.cgmlst.org/ncs

7 https://chewbbaca.online/

8 https://www.ridom.de/seqsphere/

9 https://www.applied-maths.com/applications/wgmlst

10 https://gitlab.com/bfr_bioinformatics/chewieSnake

11 https://github.com/tseemann/shovill

12 https://gitlab.com/bfr_bioinformatics/AQUAMIS

13 ftp://ftp.ncbi.nlm.nih.gov/pathogen/Results/Salmonella/

## Abbreviations

AD: allele distance
API: application programming interface
BLAST: basic local alignment search tool
CDS: coding sequence
cgMLST: core genome MLST
DAG: directed acyclic graph
DNA: deoxyribonucleic acid
HTML: hypertext markup language
INDELs: insertion and deletions
ISO: International Organization for Standardization
NCBI: National Center for Biotechnology Information
MLST: multilocus sequence typing
NGS: next-generation sequencing
SNP: single-nucleotide polymorphism
SRA: sequence read archive
ST: sequence type
wgMLST: whole-genome MLST
WGS: whole-genome sequencing.
