# Supplementary material for: Decentralized Investigation of Bacterial Outbreaks Based on Hashed cgMLST
Source: Front Microbiol. 2021 May 28;12:649517. doi: 10.3389/fmicb.2021.649517 (PMC8244591; doi:10.3389/fmicb.2021.649517)
Supplement: Supplementary file 1 [file Data_Sheet_1.docx]

Supplementary Material

# Details on workflow and software versions

The directed acyclic graphs of the two workflows can be found in the Supplementary Figure 1.

## Table of software versions and parameters

| **Scope** | **software** | **Parameter/ Version** |
| --- | --- | --- |
| **Trimming** | fastp | Version 0.19.5 |
|  |  | --detect_adapter_for_pe --n_base_limit 50 --length_required 15 |
| **Assembly** | shovill | Version 1.1.0 |
|  |  | --noreadcorr --assembler spades --depth 100 |
| **Allele calling** | chewBBACA | Version 2.0.12 |
|  |  | --st 0.2 --fr --bsr 0.6 –g **enterobase_senterica_cgmlst** --ptf prodigal_training_files/Salmonella_enterica.trn |
| **Distance and tree** | grapetree | Version 2.1 |
|  |  | -p allele_profile.tsv -m distance --missing 3 |
|  |  | -p allele_profile.tsv -m MSTreeV2 |
| **chewieSnake** | clustering | ***clustering_method: "single"***  ***distance_threshold: 10***  ***address_range: "1,5,10,20,50,100,200,1000"*** |
| **chewieSnake_join** | config | *clustering_method: "single"*  *distance_threshold: 10*  *grapetree_distance_method: 3*  *cluster_names_reservoir: chewieSnake/scripts/cluster_names_reservoir.txt*  *subcluster_distance_thresholds: [3]*  *subcluster_name_types: ['greek-alphabet']*  *external_cluster_names: NA*  *serovar_info: samples2serovars.tsv*  *do_serovar_info: True*  *species_shortname: "SE"* |

**Links to software versions and parameters**

All software versions and parameters are also described in the following files:

- Software versions: <https://gitlab.com/bfr_bioinformatics/chewieSnake/-/blob/master/envs/chewiesnake.yaml>
- chewieSnake config file: <https://gitlab.com/bfr_bioinformatics/chewiesnake_publicationdata/-/blob/master/public/chewiesnake/config_chewiesnake.yaml>
- chewieSnake_join config file: <https://gitlab.com/bfr_bioinformatics/chewiesnake_publicationdata/-/blob/master/public/chewiesnake_join/config_chewiesnake_join.yaml>

**Commands**

chewieSnake command:

*chewieSnake/chewieSnake.py -l samples_assembly_ena.tsv -d chewiesnake --scheme enterobase_senterica_cgmlst_alldata_v2 --prodigal chewieSnake/chewBBACA/CHEWBBACA/prodigal_training_files/Salmonella_enterica.trn --threads 10*

chewieSnake_join command:

*chewieSnake/chewieSnake_join.py -d . -l samples_joined_databases.tsv --serovar_info samples2serovars.tsv --species_shortname SE*

## Note on software efficiency

As described, chewieSnake is built on various modules. Allele calling – using chewBBACA – proceeds sample-by-sample and thus computational complexity grows linear with the number of samples. The time for execution varies with the amount of novel alleles, as only for these the BLASTP step is executed. Typically, the computation time is in the order of minutes. ChewBBACA itself is multithreaded, i.e. it processes loci in parallel. A higher computation time is needed for the distance matrix, minimum spanning tree and hierarchical clustering, which overall grows quadratically with the number of samples. All applied methods are tractable for substantially larger data sized as the one presented here.

# Details on the dataset and chewieSnake analysis

The data set contains 1263 sequenced Salmonella enterica WGS data and is described in more detail in <https://aem.asm.org/content/86/5/e02265-19>. The sample belong to different 74 serovars. The most prevalent serovars were Enteritidis (354), Infantis (118), Typhimurium (113),I 4,[5],12:i:- (103), Paratyphi B var. Java (69), Agona (69) and Mbandaka (62).

**Preparation of Enterobase cgMLST scheme**

Fasta files with allele sequences for every locus were downloaded from Enterobase using the web API. From the 3002 loci in Enterobase, 3000 loci can be utilized in chewBBACA, as it is required that that all loci represent a coding sequence.

The command for preparing the scheme was

*chewBBACA.py PrepExternalSchema -i referenced/Enterobase_Senterica/fasta/ --cpu 16 -v*

### Allele quality

**Overview**

All samples could be analyzed with chewieSnake. On average 97.8 % of the loci were found and an allele sequence could be identified. More than 95 % of loci were found for all, but two samples. The sample with the least fraction still contained 94.3 % of all loci - corresponding to 170 missing loci. In conclusion, sufficient targets / loci for cgMLST analysis could be found in all samples.

**Reason for missing loci**

The main reason for missing loci was that the locus was not found at all - accounting for 69% of all missing loci. 10 % of the alleles were defined as non-informative paralogous hits. 11 % and 8 % percent among all missing alleles were removed by chewBBACA as being either too long or too short respectively, compared to the reference allele sequences (compare parameter **size_threshold 0.2 in** chewBBACA).

The reason for missing loci according to chewBBACA from the analysis of thr 1263 samples read:

| LNF | PLOT | NIPH | ALM | ASM |
| --- | --- | --- | --- | --- |
| loci not found | possible loci on the tip of the query genome contigs | non-informative paralogous hit | alleles 20% larger than length mode of the distribution of the matched loci | similar to ALM but for alleles 20% smaller than length mode distribution of the matched loci |
| 0.69 | 0.01 | 0.10 | 0.11 | 0.08 |

The missing loci are largely constricted to a few specific loci. 39 loci are missing for more than 1000 of the 1263 samples and 27 loci are absent from all loci. The majority of loci (2069) are not missing in any sample.

This finding signifies that the adaptions of the Enterobase scheme to chewBBACA effectively leads to a reduction of the size of the schema by around 41 loci (corresponding to around 1% of all loci). This reduction is caused by the fact that during the allele calling step, chewBBACA cannot properly identify coding sequence for these loci.

For reference, the samples analyzed within Enterobase have on average 5.3 missing loci (median 1, max 286).

**Identification of novel alleles**

Overall, we found 33387 novel alleles - not previously known to the cgMLST scheme. For each sample we found on average 26.4 novel alleles, with only 17 % of the samples containing no novel allele. This finding depends on the order of execution, thus a sample that was analyzed at a later time point can contain alleles already found in samples that were analyzed before.

Nonetheless, novel alleles also occur frequently for samples analyzed at later stages (See Supplementary Figure 2) and we detected no indication for a saturation in the number of novel alleles found at the latter stages of the cgMLST analysis. In any case, it is important to remember that the execution order has no impact on the allele numbers themselves.

Thus, finding novel alleles is very common and properly treating these alleles is key for a comparable data analysis.

Novel alleles do not occur in equal measures for all loci. While there are 261 loci with no new alleles, 2151 loci exhibit between 1 and 10 novel alleles. 587 loci featured more than 10 novel alleles and 226 loci were attributed with more than 50 new alleles. For details, see Supplementary Figure 4.

Novel alleles are found more often in certain serovars (e.g. Paratyphi B, Typhimurium, Mbandaka) than others (Enteritidis, Agona, I 4,[5],12:i:-). This reflects the different mutation rate described in the literature, see the table below. In addition, the sample size, the order of execution and dataset specific biases also influence the detection of novel alleles.

| **Serovar** | **context** | **SNPs/site/year** | **DOI** |
| --- | --- | --- | --- |
| **Agona** |  | 5.7 × 10^−8^ -1.3 × 10^−7^ | e1003471-10.1371/journal.pgen.1003471. |
| **Enteritidis** |  | 2.2 × 10^−7^ | 10.3201/eid2009.131095 |
| **Typhimurium** |  | 1.49 × 10^-6^ | [10.1128/jcm.01733-15](http://doi.org/10.1128/jcm.01733-15) |
| **Typhimurium** | ST313 causing invasive infections in Africa | 1.9 × 10^−7^ | 10.1038/ng.2423 |
| **Typhimurium** | epidemic DT104 infections | 3.4 × 10^−7^ | 10.1126/science.1240578 |
| **Typhimurium** | 135a chicken farm outbreak | 1 × 10^-6^ | [10.1186/1471-2164-14-800](https://doi.org/10.1186/1471-2164-14-800) |
| **Typhimurium** | DT160 outbreak in New Zealand | 3.3–4.3 × 10^−7^ | 10.3201/eid2306.161934 |

### Number of clusters

For a distance cutoff of 10, we find that the data cluster into 170 different clusters - containing at least two samples. 439 samples do not cluster with the chosen threshold (*orphans* or *singletons*). 16 clusters contain 10 or more samples. The cluster number changes with a modification of the threshold as shown in Supplementary Figure 3.

# Notes on installation, software testing and validation

### Installation

All necessary information for a successful installation can be found in the repository’s README (https://gitlab.com/bfr_bioinformatics/chewieSnake/-/blob/master/README.md). For installation, one can choose between installing the conda package, a docker conainer or cloning the repository and installing dependencies using the conda package manager.

### Testing

Installation provides users with small test data for *chewieSnake* and *chewieSnake_join*.

After defining the paths of the files, e.g. by running the scripts *create_sampleSheet.sh* and *create_alleledbSheet.sh*, respectively, the wrapper scripts *chewieSnake.py* and *chewieSnake_join.py* can be run for the analysis of the test data.

See also the README for a full documentation (https://gitlab.com/bfr_bioinformatics/chewieSnake/-/blob/master/README.md).

## Validation

Validation of the results of a local installation can be performed on the test data, as well as the entire (or parts) of the data set from this contribution.

For doing so, run chewieSnake on the database of your choice. The resulting distance matrix then needs to be compared against the distance matrix provided from the test data in the repo (<https://gitlab.com/bfr_bioinformatics/chewieSnake/-/blob/master/testdata/chewiesnake/results/distance_matrix.tsv> ) or the supplementary data (full dataset, <https://gitlab.com/bfr_bioinformatics/chewiesnake_publicationdata/-/blob/master/public/chewiesnake/distance_matrix.tsv>). The assemblies to all files are available under <https://zenodo.org/record/4338293>.

### Validation of the distance matrix

The comparison can be facilitated using the provided script compare_distancematrices.R:

scripts/compare_distancematrices.R --matrix1 DISTANCEMATRIX_1 --matrix2 DISTANCEMATRIX_2 --outdir OUTDIR

The script expects two distances matrices of the same form (e.g. both either tab or space separated). See scripts/compare_distancematrices.R --help for all options.

It outputs two files

- distance_comparison_table_all.tsv: All pairwise distances with matching samples from both distance matrices
- distance_comparison_table_subset_belowthreshold_withmethoddifference.tsv: Subset of all distances that are below the defined threshold and that are non-zero

### Validation of the clustering

For the clustering comparison, a (set of) threshold needs to be defined.

For the validation of clustering, the cluster association of the samples can be found in <https://gitlab.com/bfr_bioinformatics/chewiesnake_publicationdata/-/blob/master/public/chewiesnake/cluster_addresses.tsv>

Alternatively, e.g. when using a different method or workflow the clustering can be computed with the provided script *Clustering_DistanceMatrix.R* which takes a distance matrix and a set of clustering thresholds as input (see  *Clustering_DistanceMatrix.R --help* for more information).

For estimating the clustering congruence, the online tool <http://www.comparingpartitions.info> can be applied. It requires a tsv file with a clustering from different methods and thresholds in each column.

# Supplementary Data

The Supplementary File 2 (supplementary_data_tables.zip) contains the following data tables in tsv format:

- allele_profile_comparison_repeatanalysis.tsv: File that compares allele differences and differences in missing loci between
- cluster_addresses_all_10.tsv: Cluster numbers for all methods with clustering threshold at 10 AD and 17 SNPs, respectively. This data has been used for the computation of the adjusted Wallace coefficient. Note however that the online tool cannot handle NAs, and therefore each method combination must be uploaded separately.
- pairwisedistances_enterobasecomparison.tsv: All pairwise chewieSnake and Enterobase allele distances
- pairwisedistances_fullvssplit.tsv: All pairwise chewieSnake and chewieSnake_join allele distances
- pairwisedistances_ncbisnp.tsv: All pairwise chewieSnake allele and NCBI SNP distances
- splitinformation_decentralized_analysis.tsv: Information which files can be found in which split for the chewieSnake_join analysis

The Supplementary File 3 (enterobase_senterica_cgmlst_clean.zip) contains the Enterobase cgMLST scheme used for the analyses (prior to the analysis, i.e. without any added novel alleles).
