# Supplementary material for: Decentralized Investigation of Bacterial Outbreaks Based on Hashed cgMLST
Source: Front Microbiol. 2021 May 28;12:649517. doi: 10.3389/fmicb.2021.649517 (PMC8244591; doi:10.3389/fmicb.2021.649517)
Supplement: Supplementary file 3 [file Image_1.PDF]

A

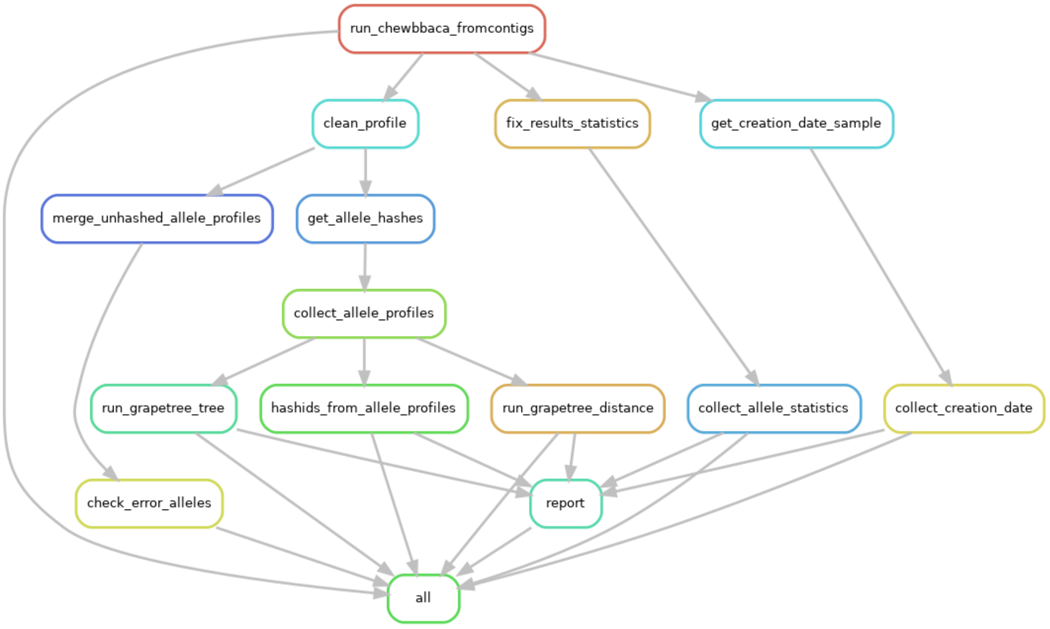

B

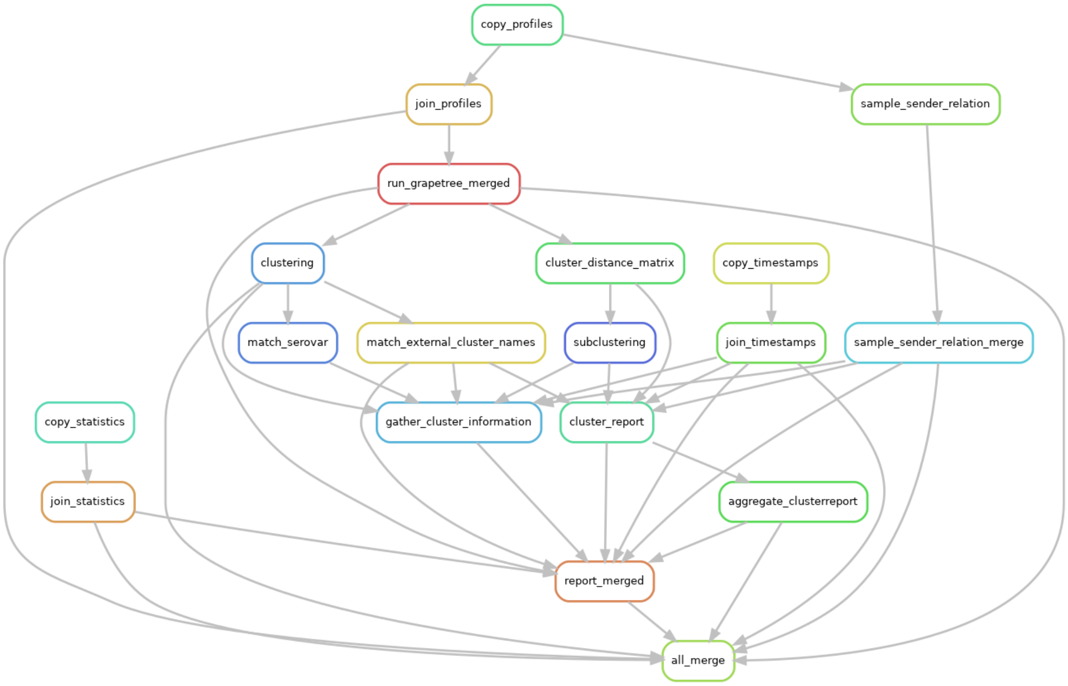

Supplementary Figure 1. : Directed acyclic graph (DAG) of chewieSnake (A) and chewieSnake_join (B) workflow. The DAG shows how the different rules are connected in Snakemake to produce the final result. Rules that are called more than once (e.g. for each sample) are only shown once.
