# Supplementary material for: Decentralized Investigation of Bacterial Outbreaks Based on Hashed cgMLST
Source: Front Microbiol. 2021 May 28;12:649517. doi: 10.3389/fmicb.2021.649517 (PMC8244591; doi:10.3389/fmicb.2021.649517)
Supplement: Supplementary file 4 [file Image_2.pdf]

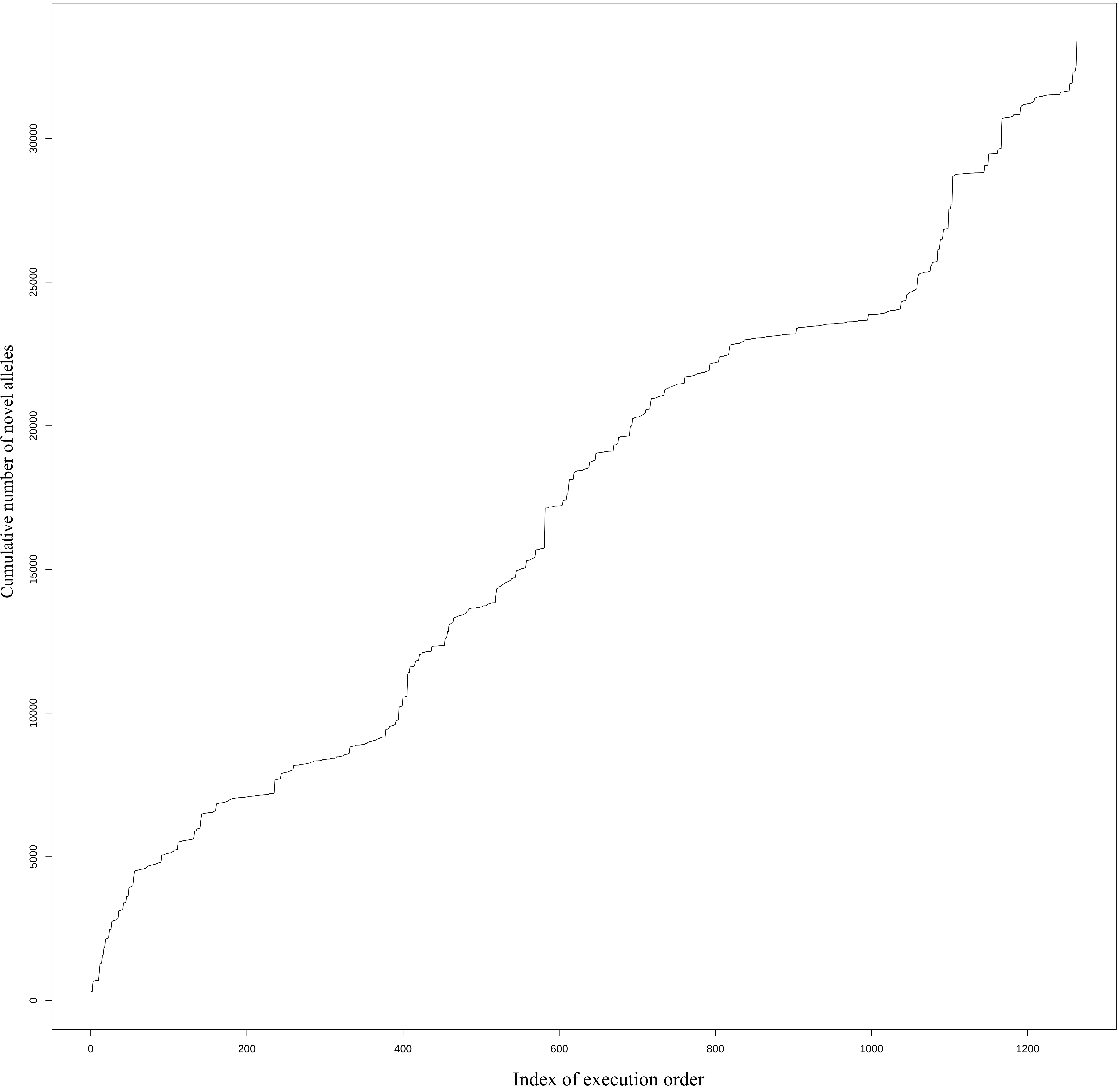

Supplementary Figure 2: Cumulative count of novel alleles. Novel alleles identified in each sample are summed in the order the allele calling was performed for each sample. The curve shows no indication of saturation, demonstrating that dealing with novel alleles is a major requirement for proper allele calling.
