# Supplementary material for: Decentralized Investigation of Bacterial Outbreaks Based on Hashed cgMLST
Source: Front Microbiol. 2021 May 28;12:649517. doi: 10.3389/fmicb.2021.649517 (PMC8244591; doi:10.3389/fmicb.2021.649517)
Supplement: Supplementary file 5 [file Image_3.pdf]

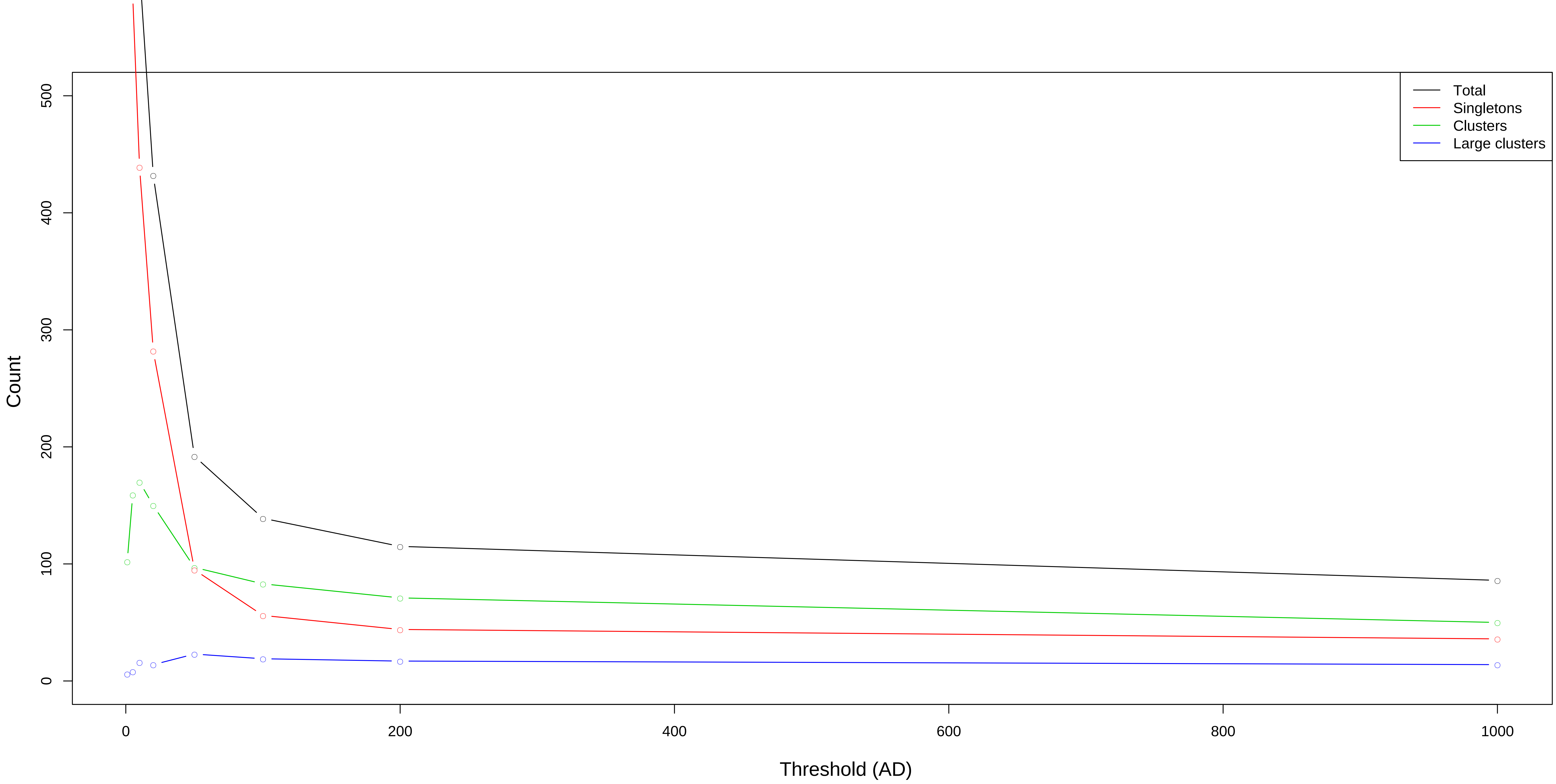

Supplementary Figure 3: Number of identified clusters for varying cluster threshold values (from chewieSnake analysis on 1263 samples). Black: Total clusters (including singletons), Red: Singletons, Green: Clusters with more than one sample, Blue: Large clusters with more than ten samples. At a threshold of 10 AD, the highest number of clusters is found. At very small thresholds, less clusters and more singletons are identified, and at large thresholds many samples end up in the same cluster.
