# Supplementary material for: Decentralized Investigation of Bacterial Outbreaks Based on Hashed cgMLST
Source: Front Microbiol. 2021 May 28;12:649517. doi: 10.3389/fmicb.2021.649517 (PMC8244591; doi:10.3389/fmicb.2021.649517)
Supplement: Supplementary file 6 [file Image_4.PDF]

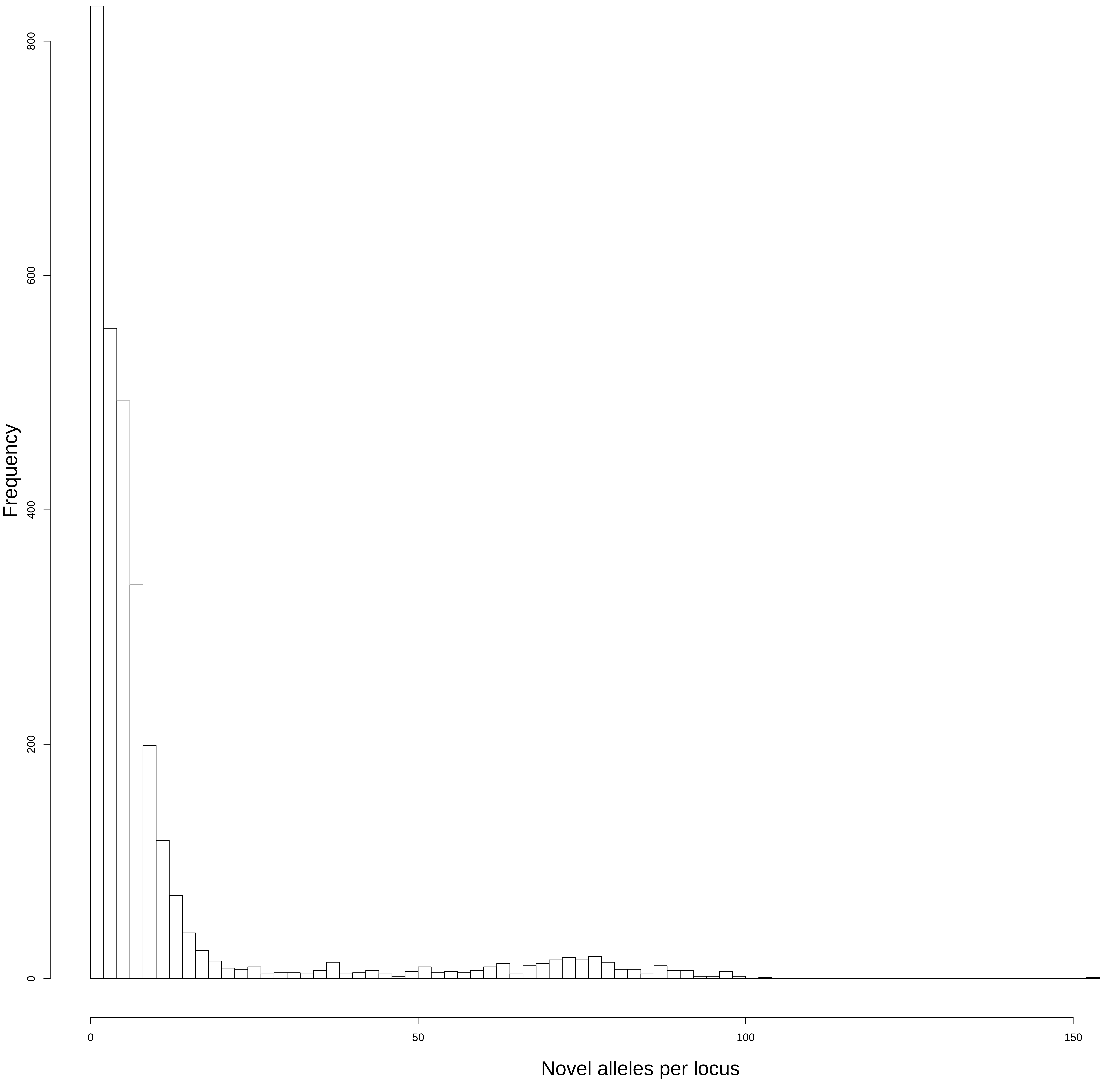

Supplementary Figure 4: Histogram of novel alleles found per locus after chewieSnake analysis on 1263 samples. While there are 261 loci with no new alleles, 2151 loci exhibit between 1 and 10 novel alleles. 587 loci featured more than 10 novel alleles and 226 loci were attributed with more than 50 new alleles. Thus, the majority of loci have relatively few novel alleles, contrasted by a fraction of the loci that evolve more dynamically.
