# Supplementary material for: Decentralized Investigation of Bacterial Outbreaks Based on Hashed cgMLST
Source: Front Microbiol. 2021 May 28;12:649517. doi: 10.3389/fmicb.2021.649517 (PMC8244591; doi:10.3389/fmicb.2021.649517)
Supplement: Supplementary file 7 [file Image_5.PDF]

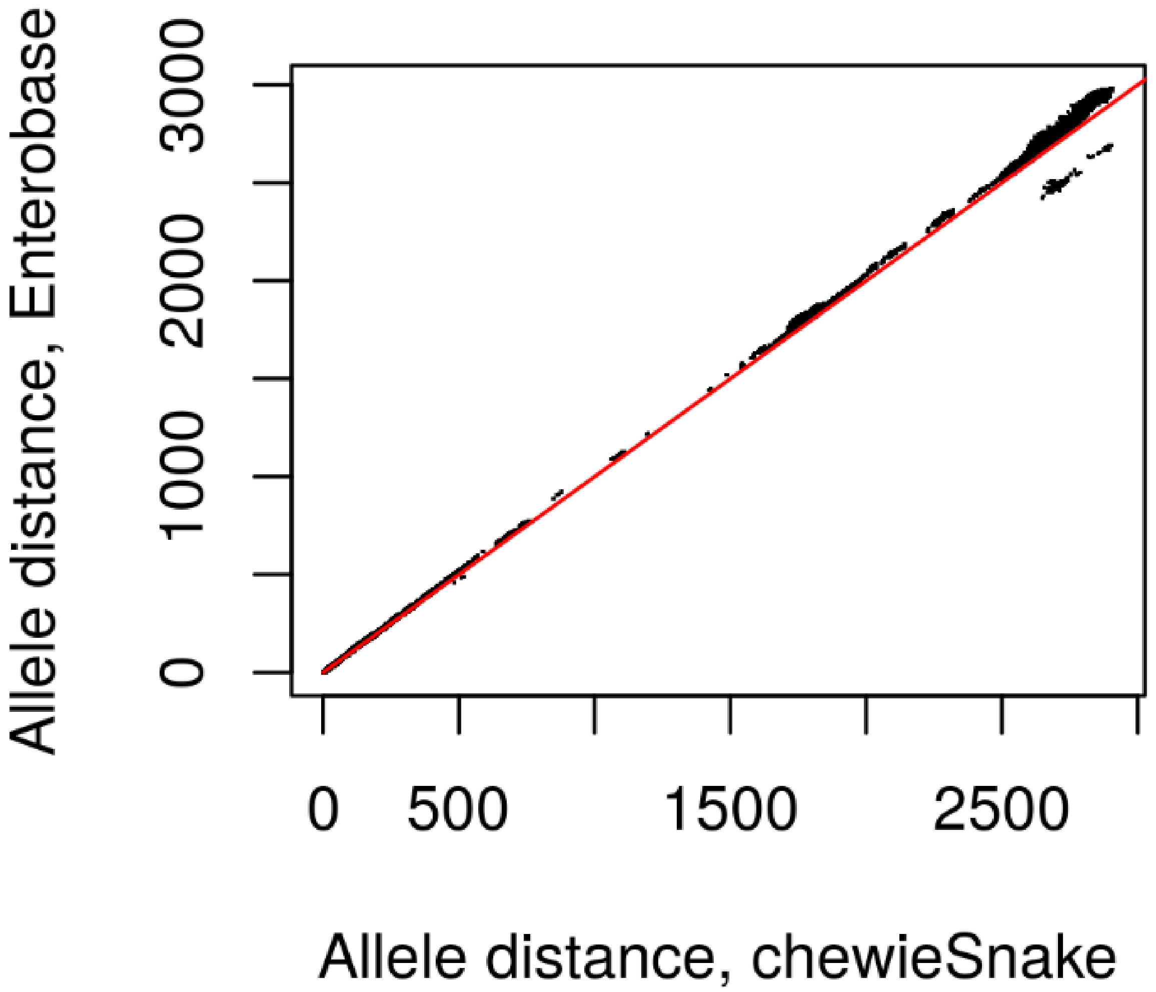

Supplementary Figure 5: Comparison of pairwise allele distances for chewieSnake and Enterobase for all commonly identified samples. The distances are strongly correlated throughout all distances and a linear fit reveals a slope of 1.02.
