# Supplementary material for: Decentralized Investigation of Bacterial Outbreaks Based on Hashed cgMLST
Source: Front Microbiol. 2021 May 28;12:649517. doi: 10.3389/fmicb.2021.649517 (PMC8244591; doi:10.3389/fmicb.2021.649517)
Supplement: Supplementary file 8 [file Image_6.PDF]

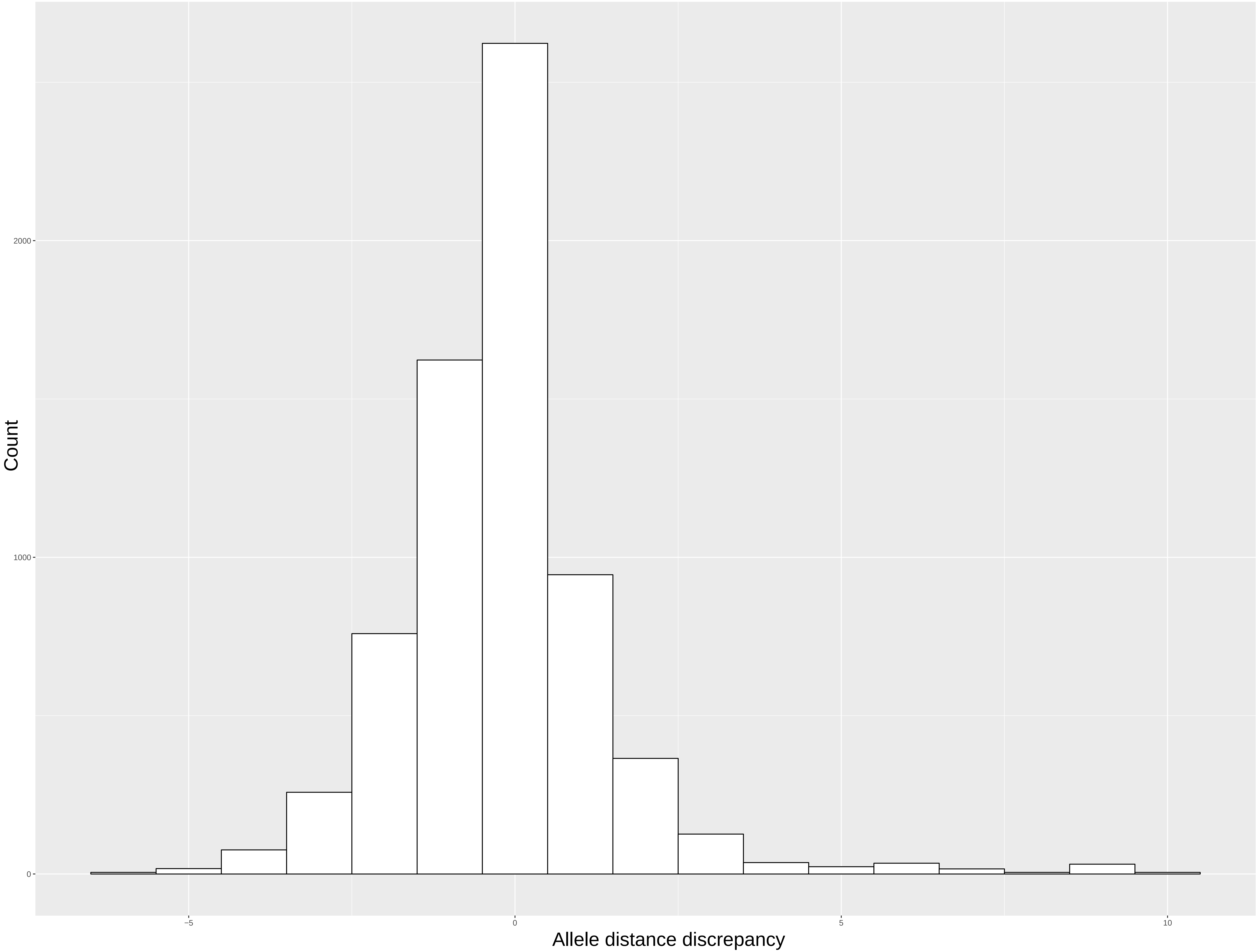

Supplementary Figure 6: Histogram of allele distance discrepancy between chewieSnake and Enterobase. All pairwise distances within 20 AD are considered for this analysis. Negative values denote a higher allele distance in Enterobase and positive values in chewieSnake. Most sample pairs have the same distance in both methods or a discrepancy of 1 AD. Also, distances in Enterobase tend too be slightly larger.
